# Special Issue: Eye-Tracking Monitoring of Neurological and Psychiatric Conditions Across Life Span

**DOI:** 10.3390/brainsci16090960

**Published:** 2026-09-11

**Authors:** Marco Cavallo

**Affiliations:** Department of Theoretical and Applied Sciences, Research Centre for Applied Psychology, eCampus University, 22060 Novedrate, Como, Italy; marco.cavallo@uniecampus.it

There is something almost paradoxical about eye movements. They are among the most conspicuous actions produced by the nervous system, yet most of the time we do not experience them as actions at all. We simply look. A fixation, a saccade, a corrective glance, or a hesitation at the edge of a visual scene seems trivial until one asks a more difficult question: what does the brain have to do to look at something, rather than merely to see it? The answer encompasses visual selection, attention, prediction, inhibition, working memory, decision making, language, motor planning and the continuous monitoring of the environment. It is this convergence of functions that makes the eye a particularly attractive, albeit imperfect, window onto brain function.

Eye tracking has consequently moved well beyond its traditional role as a tool for studying visual perception. In neurological and psychiatric research, gaze behaviour is increasingly regarded as a measurable phenotype of distributed brain dysfunction. Saccadic latency and accuracy, antisaccade errors, fixation duration, smooth pursuit, visual exploration and gaze allocation can provide indirect information about the integrity of frontal, parietal, basal ganglia, cerebellar and brainstem networks. Recent systematic evidence suggests that several of these measures may have transdiagnostic relevance, particularly for attention and executive control, although the field is still some distance from producing clinically actionable biomarkers. The attraction is obvious. Eye tracking is non-invasive, temporally precise and affordable. More importantly, it can register behaviour that occurs before a person has consciously articulated what they are doing. A person may answer correctly while taking a surprisingly inefficient route to the answer; the eyes can reveal that route. Conversely, a person may fail a conventional task while displaying an apparently appropriate pattern of visual orienting.

Neuropsychological assessment traditionally privileges overt performance, such as accuracy, reaction time, recall and errors. Eye tracking offers another layer: the dynamics by which performance is assembled. Yet precisely here lies the problem. A longer fixation is not, in itself, a cognitive deficit; an increased number of saccades is not synonymous with attentional impairment; looking at a distractor is not necessarily equivalent to processing it. Eye movements are multiply determined. Their meaning depends on the task, the stimulus, the individual’s age, visual abilities, motor status, motivation, medication, fatigue, and the cognitive operation being engaged. The recent literature is therefore characterised by a productive tension between technological sophistication and interpretative caution.

The papers collected in this Special Issue illustrate this tension across the life span. Contributions 1 and 3 suggest that in children with neurodevelopmental conditions, eye tracking can reveal the temporal unfolding of attention and language in circumstances in which conventional behavioural measures provide only the final outcome, and recent evidence suggest the potential role of this technique beyond the “standard” diagnostic process in the rehabilitation domain (contribution 6) and in vascular coupling with functional neuroimaging assessment (contribution 7). Similar principles become apparent in neurodegenerative diseases. As reported in contribution 4, in multiple sclerosis cognitive impairment is common but heterogeneous, and may be difficult to capture through brief conventional screening. Antisaccade latency and antisaccade errors seem to be among the most promising candidate metrics, while at the same time exposing the methodological fragmentation of the literature. Different devices, sampling rates, paradigms, preprocessing procedures and outcome measures make ostensibly similar findings difficult to compare. The important message is therefore not that an antisaccade metric is already a digital biomarker, but that it may become one if measurement and interpretation are sufficiently standardised. At the other end of the life span, the challenge is arguably even more compelling. In dementias, alterations in visual exploration may reflect changes in attention, executive control or visuospatial processing, but eye tracking alone may provide only part of the signal. A potentially fruitful approach can combine linguistic, behavioural and gaze measures and show that converging information across domains may provide a more coherent characterisation of cognitive impairment than any single measure considered in isolation (contribution 2). Recent systematic work in Alzheimer’s disease and mild cognitive impairment similarly suggests promise for antisaccade and visual-exploration measures, while highlighting the continuing shortage of biologically precisely characterised cohorts [1,2]. Lastly, as suggested by the contribution 5, regarding the psychiatric domain, eye tracking may be particularly informative when psychopathology is conceived in terms of cognitive and affective processes rather than diagnostic labels alone. For example, borderline personality disorder can be associated to alterations in social attention, oculomotor inhibition and autonomic responses, pointing towards a pattern in which early vigilance to socially salient information may coexist with reduced sustained engagement. This fits with a broader transdiagnostic literature showing that several eye-tracking measures of attentional control recur across psychiatric conditions, suggesting that the most useful future biomarkers may map onto dimensions such as inattention, inhibition and affective salience rather than onto traditional diagnostic categories [3,4]. Taken together, these contributions illustrate a change in emphasis. Eye tracking is no longer being used only to ask whether patients look differently. Increasingly, researchers are asking what those differences mean, when they emerge, how they evolve, and whether they can be modified. The transition from measurement to intervention is particularly important. If gaze behaviour reflects the interaction between oculomotor and higher-order cognitive systems, then the eye may become not merely a biomarker of dysfunction but also a route through which behaviour can be trained. The paediatric rehabilitation literature is beginning to explore precisely this possibility [5,6], although the evidence remains heterogeneous and longitudinal demonstrations of transfer to everyday functioning are still limited.

The major gap in knowledge, therefore, is no longer simply whether eye tracking can distinguish patients from healthy individuals. It is whether we can establish the neurocognitive meaning of a particular ocular-motor phenotype, determine whether it precedes or follows clinically observable impairment, and establish whether it changes in predictable ways with development, disease progression or treatment. We still lack sufficiently large normative datasets spanning childhood, adulthood and later life; longitudinal studies remain relatively scarce; and differences in hardware, calibration, sampling frequency, preprocessing, area-of-interest definitions and analytical pipelines continue to constrain reproducibility.

Future research should consequently move beyond the search for isolated “markers”. First, lifespan-oriented longitudinal studies are needed, because cross-sectional group differences cannot establish whether an oculomotor abnormality is a trait, a state, a compensatory mechanism or a consequence of disease. Second, eye tracking should be systematically integrated with neuropsychological, behavioural, neuroimaging, electrophysiological and biological measures. The emerging evidence suggests that convergence across modalities is likely to be more informative than the performance of any single metric. Third, ecological validity should become a methodological priority. At-home, mobile and webcam-based eye tracking, together with virtual and interactive environments, offer the possibility of measuring cognition in richer contexts and of monitoring change repeatedly rather than at a single laboratory visit. Finally, the field should embrace a transdiagnostic and computationally informed perspective. The same saccadic error may arise from different mechanisms, while apparently different gaze phenotypes may reflect a common disturbance of attentional control. What matters, therefore, is not simply the statistical separation of groups, but the identification of the latent cognitive processes that generate individual patterns of gaze behaviour.

The eye is not a transparent window onto the brain. It is a moving instrument through which the brain reveals, imperfectly but continuously, how it selects the world. The collective message of the papers published in this Special Issue is perhaps both more modest and more consequential than the promise of a new diagnostic test: eye tracking may help us understand how cognition fails, adapts and recovers, rather than merely whether a person succeeds or fails. The field will advance when reliable scientific evidence gives way to reproducible, biologically grounded, longitudinal models that explain individual trajectories and predict clinically meaningful outcomes. The intriguing promise of eye tracking is not that the eyes can “diagnose” brain’s deficits. It is that, by watching where a person looks, for how long, and what makes their gaze change, we may obtain a fine-grained view of how the changing brain visually engages with the world.
